# Movement disorders are linked to TDP-43 burden in the substantia nigra of FTLD-TDP brain donors

**DOI:** 10.1186/s40478-023-01560-7

**Published:** 2023-04-12

**Authors:** Luigi Fiondella, Priya Gami-Patel, Christian A. Blok, Annemieke J. M. Rozemuller, Jeroen J. M. Hoozemans, The Netherlands Brain Bank, Yolande A. L. Pijnenburg, Marta Scarioni, Anke A. Dijkstra

**Affiliations:** 1 Alzheimer Center Amsterdam, Neurology, Vrije Universiteit Amsterdam, Amsterdam UMC location VUmc, De Boelelaan 1118, Amsterdam, 1081 HZ The Netherlands; 2 Amsterdam Neuroscience, Neurodegeneration, Amsterdam, The Netherlands; 3 Department of Biomedical, Metabolic, and Neural Sciences, University of Modena and Reggio Emilia, Modena, Italy; 4 Department of Pathology, Amsterdam University Medical Centers, Amsterdam Neuroscience, Amsterdam, The Netherlands; 5 Swammerdam Institute for Life Sciences, University of Amsterdam, Amsterdam, The Netherlands; 6 Netherlands Institute for Neuroscience, University of Amsterdam, Amsterdam, 1105 BA The Netherlands; 7 Department of Neurology, Ghent University Hospital, Ghent, Belgium

**Keywords:** Frontotemporal Dementia, Frontotemporal Lobar Degeneration, TDP-43, Substantia nigra, Movement disorders, Brain donors

## Abstract

Movement disorders (MD) have been linked to degeneration of the substantia nigra (SN) in Parkinson’s disease and include bradykinesia, rigidity, and tremor. They are also present in frontotemporal dementia (FTD), where MD have been linked to frontotemporal lobar degeneration with tau pathology (FTLD-tau). Although MD can also occur in FTLD with TDP-43 pathology (FTLD-TDP), the local pathology in the SN of FTLD-TDP patients with MD is currently unexplored. The aims of this study are to characterize the frequency and the nature of MD in a cohort of FTLD-TDP brain donors and to investigate the relationship between the presence of MD, the nigral neuronal loss, and the TDP-43 burden in the SN. From our cohort of FTLD-TDP patients (n = 53), we included 13 donors who presented with MD (FTLD-MD+), and nine age-sex matched donors without MD (FTLD-MD-) for whom the SN was available. In these donors, the TDP-43 burden and the neuronal density in the SN were assessed with ImageJ and Qupath software. The results were compared between the two groups using T-test. We found that the TDP-43 burden in the SN was higher in FTLD-MD+ (mean 3,43%, SD ± 2,7) compared to FTLD-MD- (mean 1,21%, SD ± 0,67) (p = 0,04), while no significant difference in nigral neuronal density was found between the groups (p = 0,09). 17% of FTLD-TDP patients developed MD, which present as symmetric akinetic-rigid parkinsonism or CBS. Given the absence of a significant nigral neuronal cell loss, TDP-43 induced neuronal dysfunction could be sufficient to cause MD.

## Introduction

Frontotemporal dementia (FTD) is an umbrella term for a group of neurodegenerative syndromes that represent the second leading cause of early onset dementia, involving patients under the age of 65. The clinical presentation is heterogeneous and ranges from changes in behavior and social conduct to language and speech disturbances. Based on the predominant features, two main clinical subtypes are described: behavioral variant of FTD (bvFTD) and primary progressive aphasia (PPA), which is subdivided into semantic variant (svPPA) and nonfluent variant (nfvPPA) [1, 2]. In addition, FTD patients often develop concurrent motor features such as movement disorders (MD) [3]. MD in FTD include bradykinesia, akinesia, postural instability, tremor, rigidity, and involuntary dyskinetic movements [4, 5].

Not surprisingly, the underlying FTD neuropathology is also heterogeneous. All patients show a relatively consistent frontal and temporal lobar degeneration (FTLD), which can be further subclassified into three major groups according to the presence of aggregated proteins: TAR DNA-binding protein 43 (TDP-43), tau, and fused in sarcoma (FUS). Among them, FTLD with TDP-43 inclusions (FTLD-TDP) is the most common molecular subgroup, accounting for almost 50% of all FTLD cases [6, 7].

The pathological correlate of MD in FTD remains relatively unexplored. The frequency of MD in FTD patients is variable across the cohorts (12,5-30%) and MD are more typically linked to tauopathy [8, 9]. In Parkinson’s disease (PD), the neurobiological correlate of MD is the dopaminergic degeneration in the substantia nigra (SN) [10, 11]. In FTLD-tau donors, MD have been associated with a variable regional tau protein burden, particularly in the SN [12], while the relationship with the degree of nigral neuronal density has not been investigated.

In our previous clinicopathological study, we identified MD in FTLD-TDP donors [7], confirming that MD are widespread in the whole FTLD spectrum [13]. However, in FTLD-TDP donors, the possible role of TDP-43 deposition in the SN has not been clarified yet.

The aims of our study are to establish the frequency and nature of MD in a cohort of sporadic and genetic post-mortem FTLD-TDP brain donors and, secondly, to determine whether there is an increased burden of TDP-43 pathology or neuronal loss in the SN of FTLD-TDP donors with and without MD.

## Materials and methods

### Subjects

Post-mortem brain tissue was obtained from the Netherlands Brain Bank [14]. All donors had provided informed consent for autopsy and the use of medical records for research purposes.

In this retrospective *post-mortem* study, we analyzed all the medical records of the patients diagnosed with FTLD-TDP from the NBB (n = 53). Among them, we identified 13 patients with reported MD (rigidity, tremor, bradykinesia, postural instability, dystonia, chorea, and myoclonus) during the first three years of the disease course. Four cases were excluded due to overlapping motor symptoms, pyramidal and MD, that were difficult to differentiate. This resulted in a cohort of nine donors with MD (FTLD-MD+) and a control group of nine sex- and age- matched FTLD-TDP brain donors who never experienced MD (FTLD-MD-) and for whom SN was available.

### Clinical assessment

Two neurologists (M.S. and L.F.) with experience in FTD assessed the medical records of the donors in detail. All medical records included extensive evaluations from neurologists at the time of the diagnosis and throughout all the disease course. MD, including rigidity, tremor, bradykinesia, postural instability, dystonia, chorea, and myoclonus, were recorded individually.

Separately, when a clinical syndrome could be recognized based on the explicit mention of single symptoms in the clinical records, we evaluated the possibility of a retrospective *post-mortem* diagnosis according to the following definitions: Parkinsonism, defined as bradykinesia and at least one additional sign among resting tremor, muscular rigidity, or postural instability [15]; progressive supranuclear palsy (PSP), defined as parkinsonism with postural instability and vertical gaze abnormalities [16]; corticobasal syndrome (CBS), defined as a combination of rigidity, dystonia, or myoclonus with cortical signs [17].

### Neuropathological assessment

The brain tissue was dissected into anatomically defined structures in accordance with the Brain Net Europe’s Code of Conduct for brain banking and the declaration of Helsinki [18, 19]. In summary, the right hemisphere was fixed in 4% formaldehyde for 4 weeks, embedded in paraffin, dissected into 24 standard regions for diagnostic evaluation and cut at a thickness of 8 mm [20]. Particularly, the SN was collected at the level of the oculomotor nerve for all brain donors in our cohort. The pathological sections were evaluated by an expert neuropathologist (A. J. M. R.) according to the latest international diagnostic criteria.

All areas were immunostained for the main pathological proteins: phosphorylated-TDP-43 (pTDP43; Cosmo Bio, Tokyo, Japan), phosphorylated-tau (AT8; Pierce Biotechnology, Rockford, IL), amyloid-beta (IC16 antibody; kind gift of Prof. Dr. Korth, Heinrich Heine University, Düsseldorf, Germany), and alpha-synuclein (LB509 Thermo Fisher Scientific, Bleiswijk, The Netherlands) [21].

The pathological diagnosis relied upon clinical reports and immunostaining. Braak staging for neurofibrillary tangles (NFT) and Lewy Bodies and Thal phasing amyloid-beta plaques, phosphorylated-tau and alpha-synuclein were assessed for all brain donors [21–23]. The FTLD-TDP histotypes (FTLD Type A-E) were defined according to the criteria proposed by Lee et al. [7, 24]. Donors with TDP pathology that did not match the criteria for any TDP subtype or with overlapping features were classified as TDP-U (“unknown”).

### Nigral neuronal density quantitative assessment

We obtained high-resolution images of hematoxylin-eosin sections of the SN at the level of the oculomotor nerve from all brain donors using the Olympus VS200 digital slide scanner. In Qupath software, the SN was manually delineated for each image based on the anatomical landmarks in hematoxylin and eosin using the coordinates of the Atlas of Human Brainstem in the Ponto-Mesencephalic Junction Plane [25, 26]. The cerebral peduncles were the ventral landmarks, and the medial lemniscus the dorsal. The ventral tegmental area was included in the outline of the SN.

With Stardist, a deep-learning-based method of 2D nucleus detection that exists as a Qupath extension, the overall cell number in the SN was assessed. Once all the cells were detected, two groups of cells were manually outlined with the *Polygon tool*, generating two different classes, annotated as follows: positive cells (neuromelanin-containing neurons) and negative cells (all the remaining cells in the SN). Thereafter, an *object classifier* was created. In order to create a functional classifier, the Qupath software was trained to annotate a sufficient number of cells on the basis of the previously assigned classes. At the end of the training, the *object classifier* can correctly estimate and discriminate the neuromelanin-containing neurons from other cells. Although the *object classifier* can be saved and potentially used for all the slides, a new *object classifier* was created for each sample to compensate for any possible difference among all the images collected.

The nigral neuronal density per mm^2^ was calculated considering the numbers of neuromelanin-containing neurons and the measured area of the whole SN in each donor. We validated our quantification method in another cohort, where we compared the same donors using our method to stereological measurements [11]. We found a high correlation between the methods (r = 0.86, p > 0.001 (n = 34)), indicating that our approach provides relevant insight in neurodegeneration in the SN.

### TDP-43 pathology quantitative assessment

**Fig. 1:**
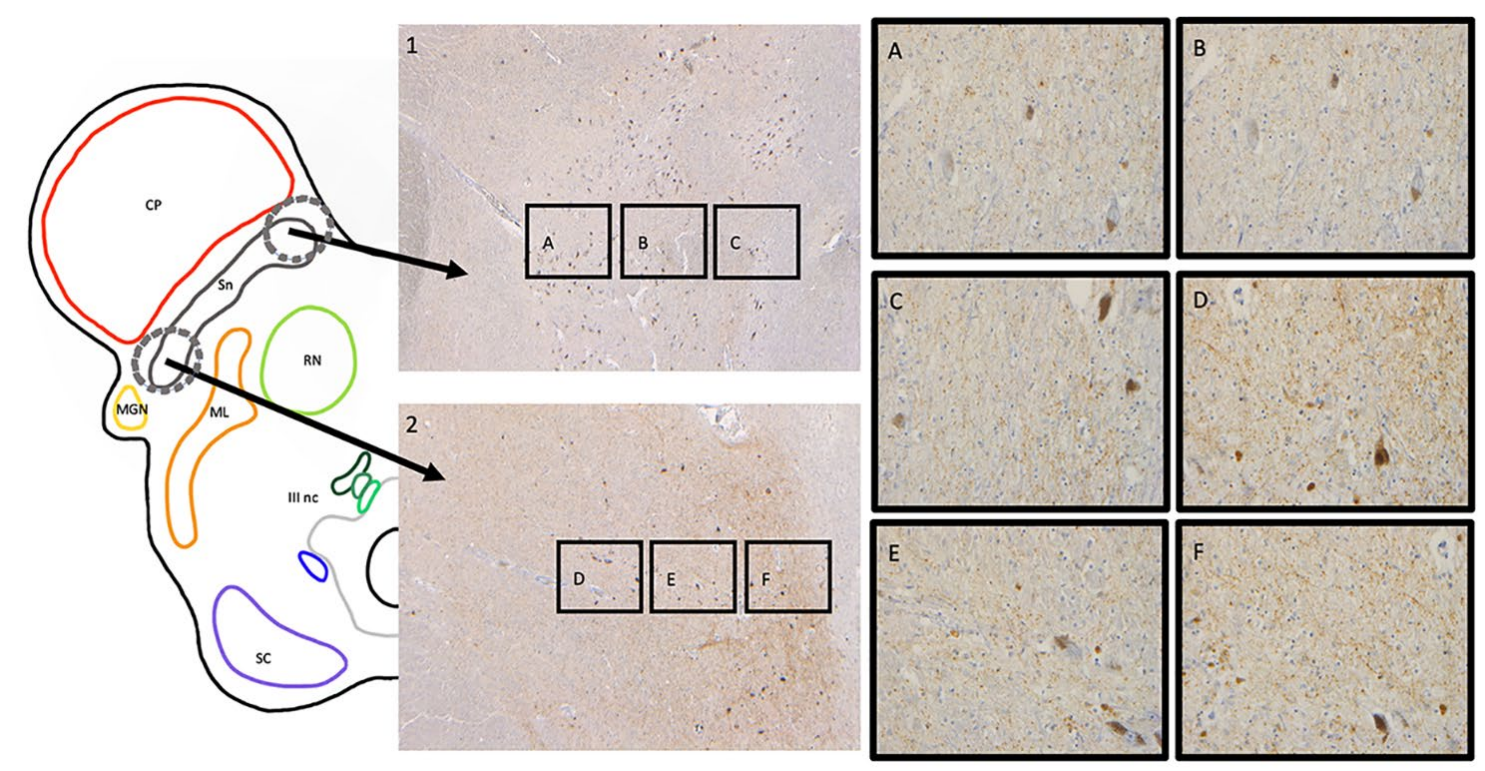
TDP-43 pathology assessment procedure The figure shows a midbrain section of the SN at the oculomotor nerve level in a FTLD-TDP type B donor. Six high-resolution pictures of ROIs in the medial (1) and the lateral (2) parts of the SN were acquired, going ventral to caudal (A-F). The pTDP-43 staining shows clear positivity in the SN, seen as small threads, granules, and neuronal cytoplasmic inclusions. A: medial ventral; B: medial central; C: medial caudal; D: lateral ventral; E: lateral central; F: lateral caudal. Abbreviations: CP: cerebral peduncle; MGN: medial geniculate nucleus; ML: medial lemniscus; Sn: substantia nigra; RN: red nucleus; SC: superior colliculus; III nc: oculomotor nucleus; ROIs: regions of interest. Scale bar: 50 μm (A-F); 400 μm (1-2)

With a quantitative technique recently validated in a previous study [27], we assessed the burden of TDP-43 pathology in the SN of FTLD-MD + and FTLD-MD-. High-resolution pictures of six regions of interest (ROIs) were acquired for each patient’s TDP-43 stained SN (N = 18). Three ROIs were drawn in the medial nigra, ventral to caudal (ventral, central, and caudal), and three ROIs were drawn in the lateral nigra, ventral to caudal (ventral, central, and caudal). Our sampling procedure is depicted in Fig. 1.

Then, each picture was processed with the “color deconvolution” plugin using ImageJ software (2019, version 1,52r) [28]. The total mean percentage of pathology was calculated for each donor using the DAB signal present in the sections. To avoid overestimation of the pathological TDP-43 burden, which has a similar color, healthy neuromelanin-containing neurons were carefully deleted manually from each picture, resulting in empty pixels.

### Statistical analysis

Mann-Whitney U test was used to compare the age of death, age of diagnosis, disease duration, Thal stages, and Braak stages between the two groups. Pearson chi-squared test was used to compare two or more categorical variables (e.g. gender distribution). The percentage of pixels showing TDP-43 deposition in the ROIs of the SN and the nigral neuronal density were compared between FTLD-MD + and FTLD-MD- using an independent sample T-test. Person correlation analysis was run for continuous variables. Statistical tests were two-tailed and a *P* value of 0.05 was considered significant. All statistical analyses were performed using the Statistical Package for the Social Sciences (SPSS version 26 for Mac, Chicago, IL).

## Results

### Demographics

Table 1 shows the demographics, clinical, and pathological information of our cohort. The median age at diagnosis (interquartile range, IQR) was 62.5 (54,75–65,5), the median age at death was 68,5 (60,75–70,5), and the median disease duration was seven years (4,75–10,25). In comparison to FTLD-MD-, FTLD-MD + donors were younger at the time of death and had a shorter disease duration. FTLD-MD + patients were older at the time of diagnosis. However, no significant differences were found with respect to gender, age at diagnosis, age at death, and disease duration between the two groups. The demographics are consistent with previous studies on the pathological cohort of FTLD donors [3, 5, 6, 29].

**Table 1:** Demographic, clinical, and pathological data

	FTDL-TDP N = 18	FTLD-MD+ N = 9	FTLD-MD- N = 9	P-value
**Demographics**				
Men, N (%)	8 (44,4)	3 (33,3)	4 (44,4)	0,629^a^
Age at diagnosis, median (IQR)	62,5 (54,75–65,5)	63 (45,5–69,5)	62 (57–63,5)	0,605^b^
Age at death, median (IQR)	68,5 (60,75–70,5)	67 (52,5–71,5)	69 (62,5–71)	0,546^b^
Disease duration, median (IQR)	7 (4,75–10,25)	7 (4–8,5)	9 (6–11,5)	0,190^b^
**Genetic status**				
C9orf72, N (%)	5 (27,7)	2 (22,2)	3 (33,3)	
GRN, N (%)	2 (11,1)	1 (11,1)	1 (11,1)	
**Clinical diagnosis**				
bvFTD, N (%)	11 (61,2)	6 (66,6)	5 (55,5)	
svPPA, N (%)	3 (16,7)	1 (11,1)	2 (22,2)	
PPA, N (%)	3 (16,7)	1 (11,1)	2 (22,2)	
CBS, N (%)	1 (5,6)	1 (11,1)	0	
**Pathological data**				
Brain weight (gr, mean, ± SD)	999,7 (± 195,14) (N 17/18)	1010,67 (± 217,17) (N 9/9)	987,37 (± 181,14) (N8/9)	0,813^c^
TDP A N, (%)	4 (22,2)	1 (11,1)	3 (33,3)	
TDP B, N (%)	9 (50)	6 (66,1)	3 (33,3)	
TDP C, N (%)	3 (16,7)	0	3 (33,3)	
TDP E, N (%)	1 (5,6)	1 (11,1)	0	
TDP U, N (%)	1 (5,6)	1 (11,1)	0	
Thal stage AB, median (IQR)	1 (0–2) (N 14/18)	0 (0–1,75) (N 8/9)	1,5 (0,75–2,25) (N 6/9)	0,228^b^
Braak stage Tau, median (IQR)	1 (0–2) (N 17/18)	1 (0,5 − 2,5) (N 9/9)	2 (0–2) (N 8/9)	0,888^b^
Braak stage LBs, median (IQR)	0 (0–0) (N 18/18)	0 (0–0) (N 9/9)	0 (0–0) (N9/9)	

Data are presented as number (%) for categorical variables and median (IQR) or mean (SD) for continuous variables. ^a^ Pearson Chi-Square. ^b^ Mann-Whitney U test. ^c^ independent samples T-test

*C9orf72* repeat expansion; *GRN* progranulin gene mutation; *bvFTD*, behavioral frontotemporal dementia; *svPPA*, semantic variant primary progressive aphasia; *PPA*, primary progressive aphasia; *CBS*, corticobasal syndrome; *TDP*, TAR DNA-binding protein 43; *LBs*, Lewy Bodies; *IQR*, interquartile range; *SD*, standard deviation

### Clinical and pathological results

#### Clinical diagnosis

The majority of the patients in both groups were diagnosed with bvFTD, with svPPA being the second most prevalent diagnosis. No difference in terms of clinical diagnosis was found between FTLD-MD + and FTLD-MD- (Pearson chi-square, p = 0,377). All the clinical diagnoses were in the FTD spectrum.

#### MD description

In the whole FTLD-TDP cohort, MD were documented in 17% of the donors (9/53). During the disease course, all donors developed rigidity, and eight donors out of nine exhibited more than one MD. Five (55,5%) patients suffered postural instability and bradykinesia. Only one (11,1%) donor experienced tremor and only one (11,1%) other donor showed dystonia. Parkinsonism was observed in four (44,4%) donors out of nine.

The majority of donors (66,6%) presented symmetric MD and, most commonly, MD were an additional symptom associated with the main FTD clinical diagnosis. Only one case developed MD as a primary symptom.

Apraxia occurred in five donors (55,5%), and a retrospective *post-mortem* clinical diagnosis of CBS could be performed in three patients (33,3%). Vertical gaze abnormalities, myoclonus, and chorea were not found in our cohort.

Regarding the pathology, MD were more frequent in TDP-B cases (66,6%), while TDP-A, TDP-E, and TDP-U were each present in one separate donor. However, MD phenomenology did not differ among different FTLD-TDP subtypes (Pearson Chi-square, p > 0,05). Table 2 shows the MD frequency in FTLD-MD + and Table 3 (at the bottom) describes the MD in each FTLD-MD + donor individually.

**Table 2:** MD frequency in FTLD-MD+

Movement disorders	N (%) N = 9
Rigidity	9 (100)
Bradykinesia	5 (55,6)
Tremor	1 (11,1)
Postural instability	5 (55,6)
Dystonia	1 (11,1)
Apraxia	5 (55,5)
Alien Limb	2 (22,2)
**Clinical Syndromes**	**N (%)** N = 9
Parkinsonism	4 (44,4)
CBS	3 (33,3)
PSP	0 (0)

The table shows retrospective data. *CBS*, corticobasal syndrome; *PSP*, progressive supernuclear palsy

**Table 3:** Demographics, genetic, clinicopathologic data in FTLD-MD + and FTLD-MD- donors

MD+	G	Age	ad	Genetic	Path	Rig (100%)	Brady (55,5%)	Tremor (11,1%)	PI (55,5%)	Apraxia (55,5%)	AL (22,2%)	VGP (0%)	Dyst (11,1%)	Sym (66,6%)	Park (44,4%)	CBS (33,3%)	TDP(%)	NND
1	F	40	38	C9	TDP-B	+	+	-	+	+	-	-	-	+	+	-	1,6	50,57
2	F	69	65	-	TDP-B	+	+	+	+	-	-	-	-	+	+	-	8,1	37,85
3	M	78	72	C9	TDP-U	+	-	-	+	+	-	-	-	-	-	+	2,9	37,77
4	M	74	74	-	TDP-B	+	+	-	-	+	-	-	-	+	+	-	7,3	67,36
5	F	67	63	GRN	TDP-A	+	-	-	-	-	-	-	-	+	-	-	1,8	65,82
6	M	69	67	n.a.	TDP-E	+	-	-	+	+	+	-	-	-	-	+	1,1	54,89
7	F	53	44	n.a.	TDP-B	+	+	-	-	-	-	-	+	+	+	-	5,1	36,63
8	F	67	63	-	TDP-B	+	-	-	+	-	-	-	-	+	-	-	1,1	60,35
9	F	52	47	n.a.	TDP-B	+	+	-	-	+	+	-	-	-	-	+	2,0	94,08
**MD-**																		
1	M	60	54	C9	TDP-B	-	-	-	-	-	-	-	-	-	-	-	0,8	55,50
2	M	69	59	-	TDP-C	-	-	-	-	-	-	-	-	-	-	-	0,8	161,23
3	M	64	60	C9	TDP-B	-	-	-	-	-	-	-	-	-	-	-	1,3	65,36
4	F	74	71	n.a.	TDP-B	-	-	-	-	-	-	-	-	-	-	-	2,2	40,15
5	F	69	63	GRN	TDP-A	-	-	-	-	-	-	-	-	-	-	-	0,6	102,76
6	F	70	64	C9	TDP-A	-	-	-	-	-	-	-	-	-	-	-	1,0	37,57
7	M	68	62	-	TDP-C	-	-	-	-	-	-	-	-	-	-	-	1,6	85,40
8	F	72	62	-	TDP-C	-	-	-	-	-	-	-	-	-	-	-	0,4	97,48
9	F	61	55	n.a.	TDP-A	-	-	-	-	-	-	-	-	-	-	-	2,2	96,72

*Case* case number; *G* gender; *Age* age at death; *ad* age at diagnosis; *Genetic* genetic status; *C9* C9orf72 repeat expansion; *GRN* progranulin gene mutation; *Path* pathological diagnosis; *Rig* Rigidity; *Brady* Bradykinesia; *PI* postural instability; *AL* alien limb; *VGP* vertical gaze palsy; *Dyst* dystonia; *Sym* symmetry; *Park* parkinsonism; *CBS* cortical basal syndrome; *TDP (%)* TDP percentage load in SN; *NND* nigral neuronal density (number of cells per mm^2^)

#### Pathological diagnosis

**Fig. 2:**
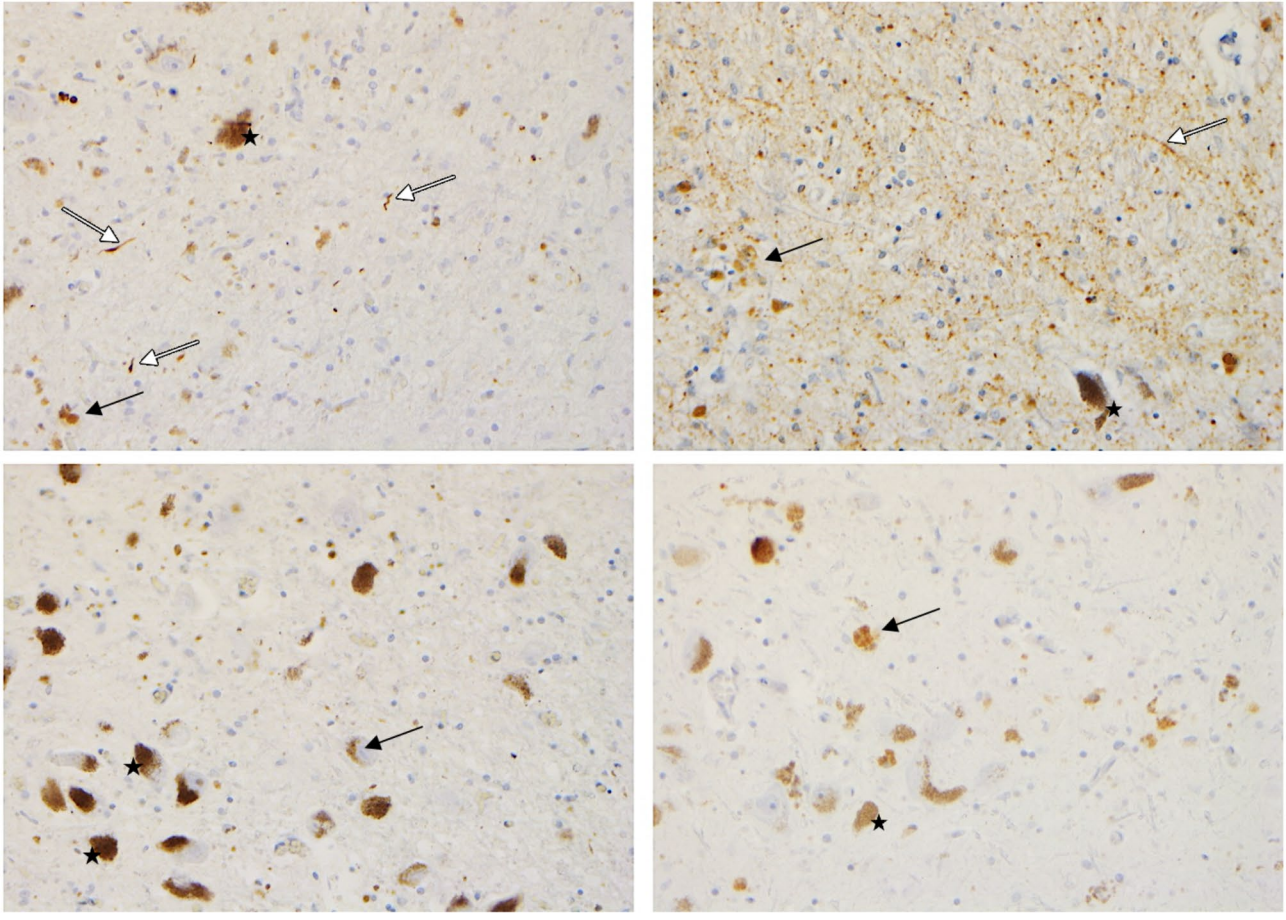
The pathological hallmark features of FTLD-TDP subtypes in the SN of brain donors from our cohort FTLD-TDP pathology is characterized by neuronal cytoplasmatic inclusions (NCI), neuronal intranuclear inclusions, and dystrophic neurites (DN). Short DN and compact NCI are seen in FTDL-TDP subtype A (top left). FTLD-TDP type B (top right) shows granular NCI and thread and dot-like pathology. The burden of local SN pathology of FTLD-TDP subtype C case (bottom left) and FTLD-type E case (bottom right) is mild, and the hallmark features of these subtypes, such as long threads and granular depositions are not present in the SN In the figure, some examples of NCI (black arrows), threads and thread and dot like pathology (white arrow) are shown. The black stars indicate some examples of neuromelanin neurons. Scale bar: 50 μm

Nine (50%) donors were classified as TDP-B, 4 (22,2%) as TDP-A, three as TDP-C (16,7%), one TDP-E (5,6%) and one (5,6%) TDP-U. In FTLD-MD+, TDP-B was the most prevalent pathological diagnosis (66,6%); whereas TDP-A, TDP-B, and TDP-C were equally distributed in FTLD-MD- (33,3%). Figure 2 shows the pathological hallmark features of FTLD-TDP subtypes in the SN of the donors in our cohort.

Eight (44,4%) donors showed amyloid co-pathology (3 FTLD-MD+, 5 FTLD-MD-), while NFT co-occurred in 12 patients (66,6%; 7 FTLD-MD+, 5 FTLD-MD-). The burden of non-TDP co-occurring pathologies -amyloid-beta and NFT- was low in all examined brain regions and none in SN. Statistical analysis revealed no significant difference in Thal phase for amyloid beta (p = 0,228) and Braak stage for NFT (p = 0, 888) between FTLD-MD + and FTLD-MD-. No alpha-synuclein pathology was found in the FTLD-TDP donors.

FTLD-MD- showed a lower cerebral weight than FTLD-MD+, although the difference was not significant.

#### Genetics

Five (27,7%) donors were positive for the *C9orf72* repeat expansion (two in the FTLD-MD + group, three in the FTLD-MD- group) and two (11,1%) donors were positive for the *GRN* mutation (one in the FTLD-MD + group and one in the FTLD-MD- group). Regarding pathology, all donors positive for the *GRN* mutation presented with the TDP-A histotype. TDP-B was the most common histotype in *C9orf72* donors (60%).

### Nigral neuronal density

The number of neuromelanin-containing neurons in FTLD-MD+ (mean 901,66; SD ± 326) was lower compared with FTLD-MD- (mean 1102,22; SD ± 639) but this was not significant (p = 0,449). The estimated mean nigral neuronal density was 56,15 (SD ± 18,58) cells per mm^2^ in FTLD-MD + and 82,46 (SD ± 38,53) cells per mm^2^ in FTLD-MD-. The mean nigral neuronal density in FTLD-MD + was lower, suggesting a possible higher degree of SN degeneration, but not statistically significant; only a trend (p = 0,09) was seen.

### TDP-43 burden in the SN

**Fig. 3:**
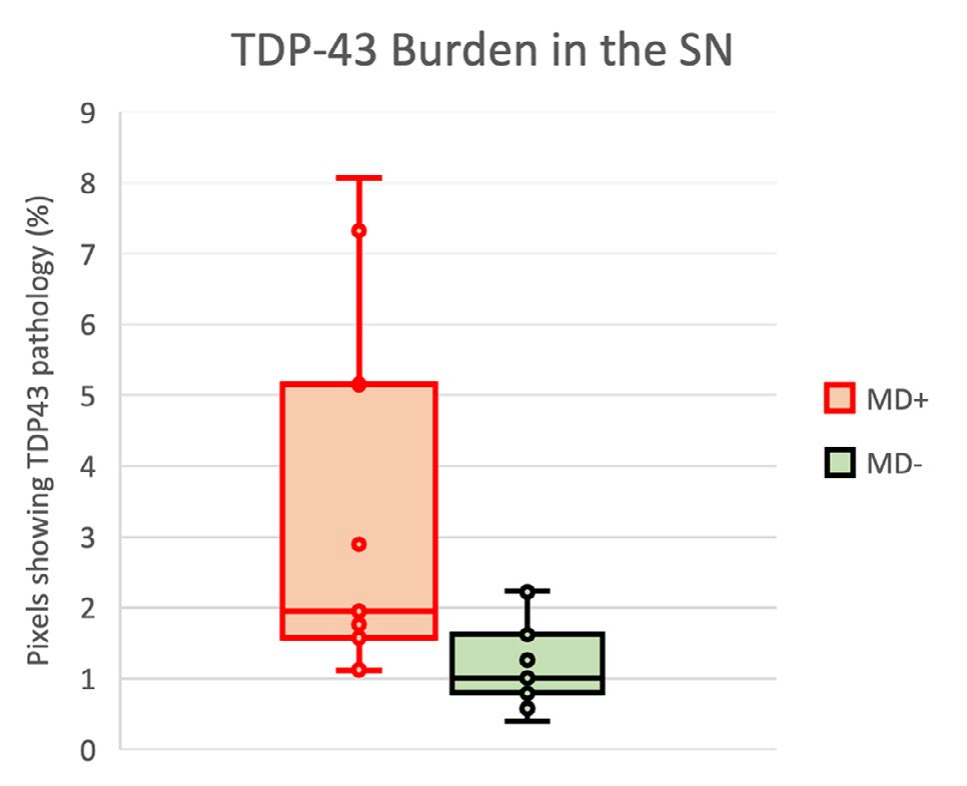
The TDP-43 burden in the SN of FTLD-MD+ and FTLD-MD- donors The total burden of TDP-43 pathology is higher in SN of FTLD-MD+ donors compared with FTLD-MD- brain donors with a statistically significant difference (p = 0,04)

Our findings revealed that the total burden of TDP-43 pathology is higher in SN of FTLD-MD + donors (mean 3,43%, SD ± 2,7) compared with FTLD-MD- brain donors (mean 1,21%, SD ± 0,67) with a statistically significant difference (p = 0,04) (Fig. 3). No correlations were found between nigral neuronal density, disease duration, and TDP-43 deposition in all patients.

Among movement disorders, we observed a higher total deposition of TDP-43 in donors who experienced rigidity and, particularly, parkinsonism. The difference was statistically significant between patients with and without rigidity (mean 3,43%, SD ± 2,7 *versus* mean 1,21%, SD ± 0,67, p < 0,05) or parkinsonism (mean 5,52%, SD ± 2,9 *versus* mean 1,4%, SD ± 0,7, p < 0,01).

## Discussion

The aim of this study was to assess the possible correlation between TDP-43 burden, neuronal density, and clinical MD. We found an increased burden of TDP-43 pathology in FTLD-TDP donors with MD compared to donors without MD, but no difference in nigral neuronal density between the groups. This suggests that the TDP-43 burden in the SN is linked to the presentation of MD in FTLD-TDP donors. In addition, we showed that MD are a prominent feature in FTLD-TDP and are therefore not solely present in FTLD-tau.

Our analysis revealed that the TDP-43 burden in the SN differs significantly between FTLD-MD + and FTLD-MD-, with FTLD-MD + having a higher burden of deposition. Previous studies showed a significant association between the presence of MD and more dystrophic neurites in the substantia nigra and midbrain tectum, with a different burden of TDP-43 distribution based on the subtype [30, 31].

However, there have been a few studies focusing on the possible relevance of regional TDP-43 concentration on clinical symptoms in FTLD which found a correlation between increased TDP-43 burden in key brain areas and specific clinical phenotypes, without any focus on MD [27, 32].

Our results point out that the TDP-43 pathology in the SN of FTLD-TDP donors contributes to the development of MD.

Interestingly, we found that the presence of MD was not associated with a higher neuromelanin-containing neuronal loss, since FTLD-MD + and FTLD-MD- did not differ significantly in terms of neuronal density in the SN. One recent study observed that SN degeneration, as determined by cell loss and gliosis with a 4 point scale semi-quantitative analysis, did not correlate with the presence of clinical symptoms, parkinsonism, or their severity [5]. A possible interpretation of our study is that the TDP-43 pathology precedes the cell loss, causing a neuronal dysfunction that results in clinical symptoms. This may imply that, independently from the SN levels of degeneration, the burden of TDP-43 pathology could be linked to a clinical phenotype with MD, particularly parkinsonism. Consistent findings on Alzheimer’s disease patients and elderly with MD showed that the presence of NFT pathology in the SN could be the mechanism by which neuronal dysfunction and MD occur, with little or no neuronal loss [33, 34].

Our results indicate that the burden of TDP-43 pathology in the SN correlates with the presence of MD in FTLD brain donors and therefore suggest that MD are associated with the burden of pathology in the SN itself, rather than a specific subtype of proteinopathy.

We presented a cohort of sporadic and genetic FTLD-TDP donors who experienced movement disorders during the disease course, which adds to the growing evidence that MD occurs in FTLD-TDP patients. Compared to FTLD-tau [3], our data revealed a lower frequency of MD in FTLD-TDP donors. However, considering that other authors presented similar results [5, 13, 24], MD could not be considered as exclusively predictive of FTLD-tau. The majority of donors showed parkinsonism, followed by CBS, but isolated MD can also be noticed. Parkinsonism was usually symmetric, appendicular, and akinetic-rigid. During the disease course, in almost all FTLD-MD + patients, MD were an additional characteristic of the FTD syndrome, mostly bvFTD, and MD was the presenting feature in only one donor. Accordingly, clinical FTD studies showed that symmetric akinetic-rigid parkinsonism is prevalent in various subtypes of FTD, especially bvFTD and nfvPPA. The presence of tremor was uncommon, although rigidity and akinesia were more widespread and MD were only occasionally the onset symptom [35]. Previous studies on clinical-genetic correlations showed similar results: a systematic review and meta-analysis on genetic FTLD concluded that parkinsonism is the most frequent movement disorder across genetic mutations (*MAPT*, *GRN*, and *C9orf72*), with no one gene being necessarily associated with a higher prevalence of parkinsonism than the others. They also report MD included rigidity, bradykinesia, postural instability, and rarely tremor, where MD were the presenting feature in 27% of the cases [36]. Therefore, the MD of our donors are consistent with the clinical and clinical-genetic cohorts of FTD patients, confirming further that in the FTLD spectrum, akinetic-rigid parkinsonism not fitting for a specific diagnosis can be present and usually an additional feature of FTD.

The vast records accessible for all FTLD-TDP donors, the detailed assessment of both clinical and pathological features, and the extensive revision of clinical and pathological diagnosis are all strengths of our study. We analyzed the burden of TDP-43 with a quantitative method, which allowed us to better estimate the amount of pathology and to avoid observer variability. Our pathology assessment approach, however, does not allow us to count and compare the number of deposits.

Another limitation of the present study is that the data’s retrospective nature may have contributed to the lack of accuracy in reporting movement disorders, which may have been underreported. Moreover, the small sample size with genetic and TDP subtype heterogeneity could have weakened the validity of our results, which should be replicated in other cohorts.

## Conclusions

To our knowledge, this is the first study to estimate the burden of pathology in the SN in FTLD-TDP donors with and without MD. We found that a higher burden of TDP-43 in SN, but not nigral neuronal loss, is linked to the clinical manifestation of MD in FTLD-TDP donors. Our work supports the notion that MD are also present in FTLD-TDP and are not solely present in FTLD-tau donors.

## Data Availability

The datasets used and/or analysed during the current study available from the corresponding author on reasonable request.
